# Vegetation cover change and classification-based zonation of Fanjing Mountain using google earth engine

**DOI:** 10.1371/journal.pone.0359961

**Published:** 2026-10-07

**Authors:** Rongrong Wang, Wengang Cui, Suihua Liu, Ting Yu, Fei Ning, Qingsong Xu, Dan Hu

**Affiliations:** 1 College of Geography and Environmental Science, Guizhou Normal University, Guiyang, China; 2 Guizhou Mountain Resources and Environmental Remote Sensing Application Laboratory, Guiyang, China; Forest Research Institute Dehradun, INDIA

## Abstract

This study utilizes the Google Earth Engine (GEE) platform, integrating Landsat and Sentinel-2 imagery from 1987 to 2022 alongside terrain data to develop a technical system for “vegetation cover inversion–classification–vertical belt spectrum analysis.” The Fractional Vegetation Cover (FVC) is estimated using a pixel dichotomy model, while spatiotemporal changes are evaluated through Theil-Sen trend analysis and Mann-Kendall tests. A multi-feature combination scheme has been designed, employing random forest models to enhance forest classification and analyze altitude zoning characteristics. The findings indicate that: (1) Over nearly 36 years, FVC in Fanjing Mountain has demonstrated a pattern of “fluctuating decline–adjustment–jump increase,” with 2007 serving as a pivotal turning point; notably, 41.65% of the area exhibited significant improvement, particularly in the southern region; (2) The classification model that integrates terrain features, red-edge index, and texture attributes (T9 scheme) achieves an overall accuracy of 77.88%, with terrain factors being the most influential in significantly enhancing the identification of shrub meadows and evergreen broadleaf forests; (3) Distinct vertical zoning of vegetation is observed: evergreen broadleaf forests dominate lower to mid-elevations (750–1550 m), deciduous broadleaf forests and coniferous forests are found at mid-to-high elevations, while shrub meadows are concentrated above 2400 m, forming a gradient succession sequence. This study provides methodological support and scientific evidence for ecological conservation efforts in subtropical mountain regions.

## 1. Introduction

Global climate change, exacerbated by human activities, has resulted in the ongoing degradation of both the structure and function of terrestrial ecosystems [1,2]. As a fundamental component of these ecosystems, vegetation plays a crucial role in influencing regional ecological security through its functions such as carbon sequestration, soil and water conservation, and biodiversity maintenance [3–6]. The current warming climate and the increasing frequency of extreme events have accelerated processes like deforestation and vegetation degradation. Mountain ecosystems, characterized by their vertical gradients, are particularly vulnerable to climate change; phenomena such as shifts in forest belts and changes in species composition have emerged as significant ecological warning signals. In this context, accurately analyzing the spatiotemporal evolution and vertical differentiation patterns of vegetation cover is essential for informing effective ecological management strategies.

Fanjing Mountain is recognized as the “ecological gene bank at 27°N latitude” [7–9]. However, in recent years, climate warming and drying, along with increased human disturbances, have resulted in blurred boundaries between forest types and disordered vertical belt spectrum within this region. Traditional monitoring methods are constrained by limitations in temporal and spatial resolution as well as the complexity of the terrain, making precise and dynamic assessments challenging. Conventional vegetation surveys depend on ground plots and manual interpretation; while these approaches yield high data accuracy, they are hindered by elevated costs, lengthy timeframes, and inadequate large-scale continuity [10]. Remote sensing technology has enabled extensive monitoring of vegetation coverage through spectral indices such as NDVI [11–15], yet the presence of clouds in mountainous environments leads to significant data loss in optical remote sensing. Furthermore, existing algorithms demonstrate insufficient adaptability to complex terrains [16]. Although multi-source data fusion techniques can partially mitigate the limitations associated with single datasets, challenges regarding spatio-temporal consistency and model robustness persist. The Google Earth Engine (GEE) platform offers a new paradigm for long-term ecological monitoring due to its PB-level remote sensing data capabilities and cloud computing advantages [17–19]. however, its potential application for optimizing mountain vegetation classification models and analyzing vertical belt spectrum remains largely unexplored. Additionally, understanding the coupling mechanisms between changes in vegetation coverage and carbon cycling alongside climate feedbacks—along with elucidating the regulatory role of forest vertical belt spectrum in energy balance—continues to be at the forefront of research endeavors [20,21]. Current studies still exhibit deficiencies related to multi-source data fusion processes, terrain correction methodologies, and quantification efforts concerning vertical belt spectrum within mountain ecosystems.

This study focuses on Fanjing Mountain as a representative area, integrating multi-source remote sensing data from platforms such as Landsat and Sentinel-2. A random forest classification model that incorporates terrain factors is developed based on the Google Earth Engine (GEE) platform, establishing a technical system comprising “coverage inversion - type classification - vertical belt spectrum analysis.” The research aims to overcome the technical challenges associated with vegetation monitoring in mountainous regions characterized by frequent cloud cover. It seeks to elucidate the driving mechanisms behind the vertical differentiation of subtropical mountain forests and provide methodological support for the conservation of ecologically fragile areas located at similar latitudes.

## 2. Materials and methods

### 2.1. Overview of the study Area

The Fanjing Mountain National Nature Reserve is situated in the northeastern region of Guizhou Province, at the transitional zone between the Yunnan-Guizhou Plateau and the Xiangxi Mountainous Region. Its highest peak, Fenghuang Mountain, reaches an elevation of 2,572 meters. The reserve boasts a distinctive geological landscape characterized by stepped and layered terrain with a vertical difference exceeding 2,000 meters, dome-shaped structural mountain ranges, and four distinct geomorphic units. The subtropical monsoon climate has fostered a complete vertical climate zone within the reserve. Additionally, moist evergreen broad-leaved forests cover 76.1% of the area, providing essential habitat for endangered species such as the Guizhou golden snub-nosed monkey. Serving as a refuge for organisms during the Quaternary ice age and as a vital water source for the middle reaches of the Yangtze River, this reserve integrates both karst and metamorphic rock ecosystems. With its 46 vegetation communities and comprehensive soil zonation spectrum, Fanjing Mountain National Nature Reserve stands out as a natural laboratory for studying biodiversity and ecological evolution. The detailed overview of the study area is shown in Fig 1.

**Fig 1:**
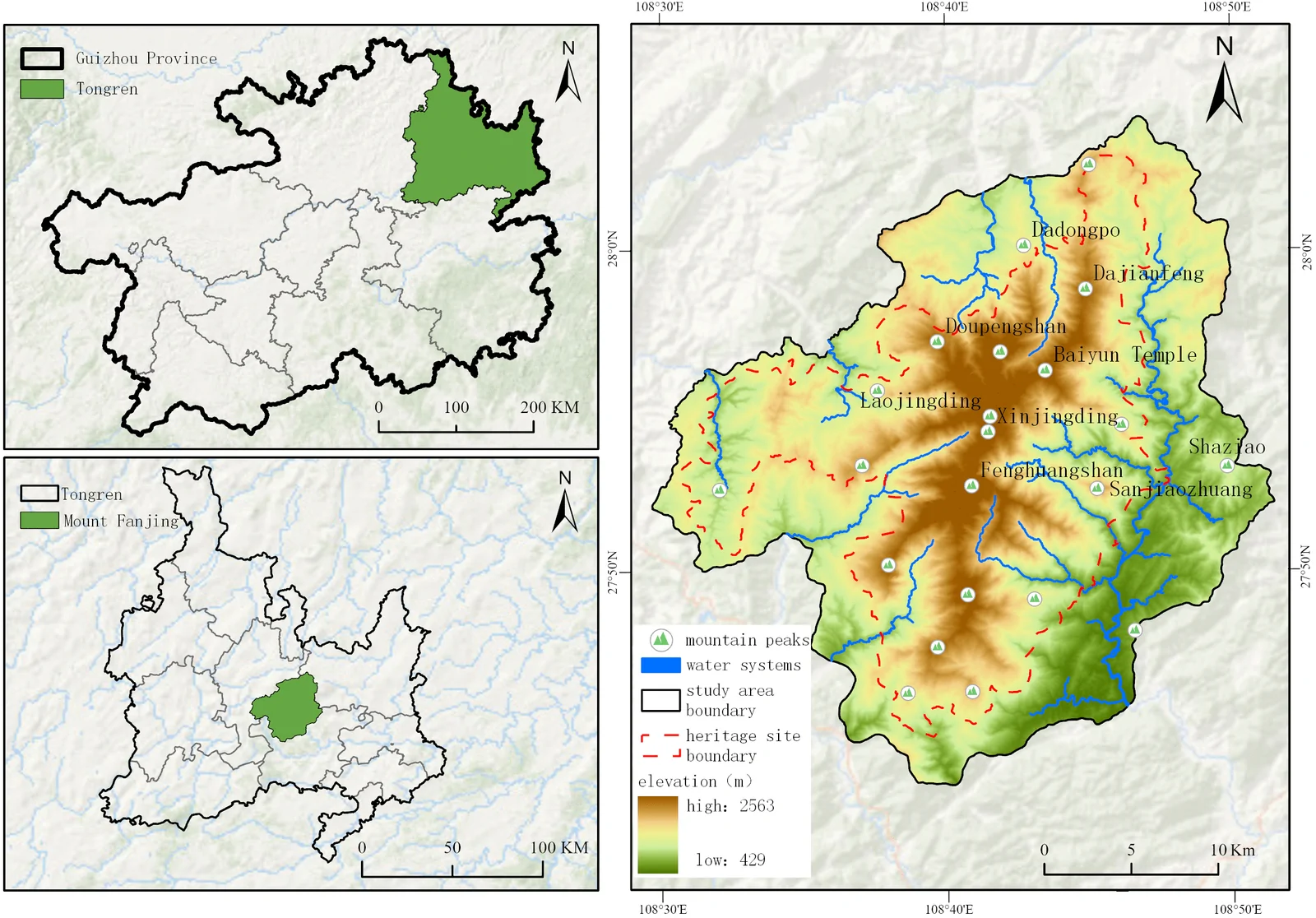
Geographical location and topographic map of The Fanjing Mountain National Nature Reserve. This figure was created with ArcGIS Pro (https://pro.arcgis.com/).

### 2.2. Data sources

This study is grounded in the analysis of multi-spectral images obtained from Landsat 5, 7, and 8, as well as Sentinel-2, alongside SRTM DEM data spanning from 1987 to 2022. The Landsat data serves as a basis for the inversion of long-term vegetation coverage, while Sentinel-2 imagery—integrated with terrain and texture features—is utilized for high-precision classification. Auxiliary data comprises forest resource surveys, unmanned aerial vehicle (UAV) aerial imagery, and vector maps delineating the functional zoning of protected areas.

#### 2.2.1. Landsat series data.

This study utilizes the standardized surface reflectance (SR) datasets from the Landsat series, which have been released by the United States Geological Survey (USGS; https://earthexplorer.usgs.gov/) and accessed via the Google Earth Engine (GEE) platform (https://developers.google.com/earth-engine/datasets). The datasets encompass Landsat 5 TM (1984–2012, dataset ID: LANDSAT/LT05/C02/T1_L2), Landsat 7 ETM+ (1999-present, dataset ID: LANDSAT/LE07/C02/T1_L2), and Landsat 8 OLI/TIRS (2013-present, dataset ID: LANDSAT/LC08/C02/T1_L2), all featuring a spatial resolution of 30 meters. Table 1 details the Landsat sensor models and their specific purposes for each time period in this study.

**Table 1:** Sensors and corresponding time periods.

Time Period	Sensor	Spatial Resolution	Main Purpose
1984–2012	Landsat 5 TM	30 m	Constructing early-to-mid-term continuous annual datasets
2012	Landsat 7 ETM+	30 m	Filling the observational gap between Landsat 5 and Landsat 8
2013–2022	Landsat 8 OLI/TIRS	30 m	Constructing recent high-precision continuous annual datasets

To mitigate the wedge-shaped banding effects present in the Landsat 7 data, the multi-temporal median composite method is employed to perform data gap filling. To ensure the validity of the multi-sensor synergistic analysis, the band reflectances of Landsat 8 are converted into equivalent radiometric values consistent with those of Landsat 5 and 7. Specifically, based on strict spectral band matching, an empirical linear transformation method utilizing Ordinary Least Squares (OLS) regression is employed to perform radiometric normalization on a band-by-band basis, thereby ensuring the reliability of the vegetation cover dynamics analysis spanning from 1987 to 2022. Furthermore, discrepancies between multi-sensor data were addressed through spectral band matching and radiometric normalization. The datasets have undergone atmospheric correction, geometric correction, and cloud removal processes conducted by USGS and are readily accessible on the GEE platform. This facilitates dynamic analysis of vegetation coverage spanning from 1987 to 2022.

#### 2.2.2. Sentinel-2 data.

This study employed the Sentinel-2 Multi-Spectral Instrument (MSI) Level-2A surface reflectance dataset (COPERNICUS/S2_SR; https://developers.google.com/earth-engine/datasets/catalog/COPERNICUS_S2_SR), provided by the European Space Agency (ESA) Copernicus program (https://dataspace.copernicus.eu/) and accessed via GEE). We selected images with cloud coverage of less than 20% during the period from May 1st to November 30th, 2020. This approach yielded a total of 11 high-quality, cloud-free datasets. The data encompass 13 spectral channels (ranging from 0.490 to 2.190 µm), with particular emphasis on the red-edge region (B5/B6/B7 bands), which significantly enhances the capability for inverting vegetation physiological parameters. The spatial resolutions are as follows: 10 meters for visible and near-infrared bands, 20 meters for red-edge and short-wave infrared bands, and 60 meters for aerosol bands. The data have undergone rigorous radiometric calibration and atmospheric correction processing, rendering them suitable for high-precision research in forest vegetation classification. Detailed specifications regarding the spectral parameter indicators of each band can be found in Table 2.

**Table 2:** Sentinel-2 Band Parameters.

Band	Band name	Central wavelength/µm	Spectral range/µm	Resolution/m
B1	Aerosol Coastal	0.443	0.433-0.453	60
B2	Blue	0.490	0.458-0.522	10
B3	Green	0.560	0.543-0.577	10
B4	Red	0.665	0.650-0.680	10
B5	Red Edge 1	0.705	0.698-0.713	20
B6	Red Edge 2	0.740	0.733-0.747	20
B7	Red Edge 3	0.783	0.773-0.793	20
B8	NIR	0.842	0.785-0.899	10
B8A	Narrow NIR	0.865	0.855-0.875	20
B9	Water Vapor	0.945	0.935-0.955	60
B10	Cirrus	1.375	1.360-1.390	60
B11	SWIR1	1.610	1.565-1.655	20
B12	SWIR2	2.190	2.100-2.280	20

#### 2.2.3. Topographic data.

The terrain data is sourced from the 30-meter resolution Digital Elevation Model (DEM) dataset (ID: USGS/SRTMGL1_003; https://developers.google.com/earth-engine/datasets/catalog/USGS_SRTMGL1_003), produced by the Shuttle Radar Topography Mission (SRTM). This dataset was acquired through dual-band radar interferometry utilizing C/X-band technology, encompassing a geographical range from 56°N to 60°S. It boasts a relative elevation accuracy of ±6 meters and offers a horizontal resolution of 1 arc-second (approximately equivalent to 30 meters), thereby delivering high-precision terrain parameters essential for analyzing surface heterogeneity.

To address the spatial resolution inconsistencies between the 20 m and 60 m bands of the Sentinel-2 multispectral imagery and the 30 m SRTM DEM data, spatial scale standardization is implemented during the data preprocessing stage. Specifically, utilizing the highest spatial resolution of the Sentinel-2 imagery (10 m, namely the visible and near-infrared bands) as the reference baseline, and operating on the Google Earth Engine (GEE) cloud platform via the bilinear interpolation algorithm, the 20 m and 60 m bands of Sentinel-2, as well as the 30 m resolution SRTM DEM data, are uniformly resampled to 10 m.

#### 2.2.4. Other auxiliary data

(1) Aerial Images: High-resolution aerial images with a resolution of 0.2 meters were acquired for the study area during the growing seasons from 2020 to 2022. Each individual image measures 5200 × 5200 pixels. These images are primarily utilized for constructing a remote sensing interpretation sample library and conducting spatial verification, thereby significantly enhancing the accuracy of forest vegetation classification.
(2) Forestry Resource Survey Data: Utilizing data from the second national forest resource inventory, a comprehensive classification system was developed in accordance with the standards outlined in the “Vegetation Map of the People’s Republic of China (1:1,000,000)” [22]. This dataset encompasses multi-dimensional feature parameters that include topographic characteristics—such as landform types, slope grade classifications, slope aspect distributions, and slope position attributes—as well as soil attribute parameters that consist of soil type classifications and effective soil layer depth measurements. Additionally, stand feature parameters are included; these encompass origin information, dominant tree species (or groups), canopy closure percentages, age classes, and volume per hectare among others. Collectively, these elements form a robust background parameter set for multi-scale forest vegetation classification.

### 2.3. Research Methods

#### 2.3.1. Vegetation coverage inversion.

Fractional Vegetation Cover (FVC), defined as the ratio of the projected area of vegetation to the total surface area [23], serves as a vital indicator for quantifying surface vegetation density, assessing growth conditions, and evaluating ecological health through remote sensing techniques. The pixel dichotomy model, a widely recognized classical method in FVC remote sensing inversion, is grounded in the theory of mixed pixel decomposition. This method operates on the core physical assumption that, constrained by the spatial resolution of remote sensing imagery, the spectral signal of an individual mixed pixel is a linear combination of pure vegetation and pure non-vegetation (e.g., bare soil) areas. By constructing a linear spectral mixture equation, the pixel’s reflectance (*R*) can be expressed as a weighted sum of the pure vegetation reflectance ($R_{v}$) and pure non-vegetation reflectance ($R_{s}$), where the weight is the FVC. Equation (1) represents the mathematical expression:


$$\begin{array}{c} R=FVC\cdot R_{v}+(\text{1}-FVC)\cdot R_{s} \end{array}$$ (1)

Solving this equation yields the foundational FVC formula (Equation 2):


$$\begin{array}{c} FVC=\frac{R-R_{s}}{R_{v}-R_{s}} \end{array}$$ (2)

To overcome the difficulty of acquiring large-scale measured reflectance data, NDVI is substituted into this model based on the spectral response mechanism of vegetation cover. Theoretically, the extreme NDVI values in endmember pixels are characterized as follows: pixels with full vegetation cover approach 1, whereas bare surfaces or non-vegetated areas approach 0. Accordingly, the FVC estimation model used in this study is formulated as Equation (3):


$$\begin{array}{c} FVC=\frac{NDVI-NDVI_{soil}}{NDVI_{veg}-NDVI_{soil}} \end{array}$$ (3)

Considering the interannual climate variability in the Fanjingshan region, residual sensor differences, and the uneven distribution of extreme pixels, the endmember parameters are dynamically calibrated on an annual basis. Specifically, using cumulative frequency statistics for the annual NDVI data from 1987 to 2022, the 5th and 95th percentiles are designated as the yearly NDVI_min and NDVI_max, respectively. This method ensures that the FVC accurately reflects the relative coverage level of the corresponding year, effectively mitigating early vegetation underestimation and spurious long-term trends caused by fixed thresholds. In comparison to regression or machine learning models, the pixel dichotomy model boasts fewer parameters, faster computation times, and reduced dependence on sample data. These attributes render it particularly suitable for large-scale and long-term dynamic monitoring applications.

The research is based on a comprehensive analysis of long-term Landsat TM/ETM/OLI series images spanning from 1987 to 2022. Utilizing the Google Earth Engine (GEE) platform, we conducted cloud removal, radiometric correction, and other preprocessing techniques to effectively mitigate the impacts of cloud contamination and atmospheric disturbances. Upon obtaining the preprocessed Landsat series imagery, the NDVI is first calculated scene-by-scene using the normalized ratio of the near-infrared and red bands. Subsequently, the Maximum Value Composite (MVC) method is applied to extract the maximum pixel-wise NDVI within each month to generate the monthly NDVI data. These monthly outputs are further composited into annual maximums, ultimately constructing the 1987–2022 annual NDVI dataset for the Fanjingshan Nature Reserve. The NDVI values were subsequently converted into Fractional Vegetation Cover (FVC) using a pixel dichotomy model. This process facilitated the construction of an annual FVC dataset from 1987 to 2022 as well as a spatial distribution map illustrating the average FVC over a span of 36 years, thereby enabling an evaluation of trends in vegetation coverage changes.

#### 2.3.2. Trend analysis method.

The Theil-Sen Median trend estimation method is a robust non-parametric statistical algorithm specifically designed for the analysis of trends in time series data. It showcases significant technical advantages when dealing with extensive time series datasets, owing to its remarkable computational efficiency and resilience against outliers. In practical applications, this algorithm is frequently integrated with the Mann-Kendall (M-K) non-parametric test to establish a comprehensive analytical framework [24]. Initially, the Sen trend estimation quantifies the intensity of trends within the time series, followed by employing the M-K test to assess the statistical significance of any observed trend changes through hypothesis testing. This synergistic analytical approach not only enhances the robustness of trend estimations but also circumvents limitations associated with assumptions about data distribution due to its non-parametric nature. Consequently, it provides a reliable dual verification mechanism for trend analysis in various fields such as ecological environment monitoring and climate change research. Its calculation formula is shown in Equation (4):


$$\begin{array}{c} \beta =median\left(\frac{X_{j}-X_{i}}{j-i}\right),\forall j>i \end{array}$$ (4)

In the formula, the slope is represented by a variable, while *j* and *i* denote the time sequence numbers. The observed values at the *j*th and *i*th time points are correspondingly indicated. A slope greater than 0 signifies an upward trend in the sequence, whereas a slope less than 0 indicates a downward trend. The Mann-Kendall test relies on comparing data ranks and does not necessitate the assumption of normal distribution. It assesses the significance of trends by constructing the statistic Z. For the time series *X_i*, where *i* = 1, 2,..., i,..., j,..., n. The calculation of the standardized test statistic *Z* relies on the sequence of formulas presented in Equations (5) to (8):


$$\begin{array}{c} \text{Z}=\left\{ \begin{array}{ccc} \frac{s-\text{1}}{\sqrt{Var(S)}} & & S>\text{0}\\ \text{0} & & S=\text{0}\\ \frac{S+\text{1}}{\sqrt{Var(S)}} & & S<\text{0} \end{array}\right. \end{array}$$ (5)

$$\begin{array}{c} S=\sum_{i=\text{1}}^{n-\text{1}}\sum_{j=i+\text{1}}^{n}sgn\left(x_{j}-x_{i}\right) \end{array}$$ (6)

$$\begin{array}{c} sgn\left(x_{j}-x_{i}\right)=\left\{ \begin{array}{ccc} +\text{1}, & & x_{j}-x_{i}>\text{0}\\ \text{0}, & & x_{j}-x_{i}=\text{0}\\ -\text{1}, & & x_{j}-x_{i}<\text{0} \end{array}\right. \end{array}$$ (7)

$$\begin{array}{c} Var(S)=\frac{n(n-\text{1})(\text{2}n+\text{5})}{\text{1}\text{8}} \end{array}$$ (8)

In the formula, n denotes the number of data points within the sequence. The variables j and i represent the data from the jth and ith time series, respectively. Here, n signifies the length of the sequence in years.

#### 2.3.3. Random forest classification.

Random Forest (RF) is an ensemble learning algorithm that consists of multiple decision trees, which collectively yield robust predictions through majority voting for classification tasks or averaging for regression tasks. The model construction process involves the following steps: 1 generating training subsets from the original dataset with replacement using the Bootstrap method; 2 randomly selecting a subset of features to determine optimal splits at each node; 3 fully developing decision trees until specified termination conditions are met; and 4 aggregating the results from each tree. The model employs Gini Impurity as a measure of node impurity and ranks feature importance to enhance variable selection.This study utilizes the Google Earth Engine (GEE) platform, employing the ee.Classifier.smileRandomForest classifier, which integrates Bagging sampling and feature randomization mechanisms to achieve high-precision classification of multi-source remote sensing images. Key parameters include the number of trees, the number of splitting variables, minimum sample size for leaf nodes, sample ratio, and random seed—ensuring both effectiveness and stability in complex mountainous environments.For any given node S, its Gini impurity is defined as Equation (9):


$$\begin{array}{c} Gini(S)=\text{1}-\sum_{k=\text{1}}^{k}p_{k}^{\text{2}} \end{array}$$ (9)

Among these, pk represents the proportion of samples of the kth type within node S, where k denotes the total number of categories.

This study is conducted on the GEE cloud platform and employs the ee. Classifier. smileRandomForest() method for remote sensing image classification. It capitalizes on the strengths of random forest algorithms in parallel computing with large datasets, while also enhancing the generalization capability of high-dimensional features through Bagging and random feature sampling. The core parameter configuration is detailed in Table 3.

**Table 3:** Parameters and explanations of the random forest model.

Parameter	Explanation
Number Of Trees	The number of decision trees: the more trees there are, the more stable the model becomes, but the computational load increases.
Variables Per Split	The number of features randomly selected at each node split, if not specified, is the square root of the total number of features.
Min Leaf Population	Minimum number of samples at leaf nodes to prevent overfitting. The default value is 1.
Bag Fraction	The proportion of samples used by each tree, with a default value of 0.5.
Max Nodes	The maximum number of nodes for a single tree, if not specified, is by default unlimited.
Seed	Random seed, ensuring reproducibility, default value is 0.

#### 2.3.4. Precision verification method.

This study utilizes the confusion matrix, generated by the errorMatrix() function in GEE, as a fundamental tool for evaluating classification accuracy. By comparing predicted categories with actual categories, it quantifies the effectiveness of classification. The confusion matrix offers key indicators such as overall accuracy (OA), producer’s accuracy (PA), user’s accuracy (UA), Kappa coefficient, and F1 score, thereby providing a comprehensive assessment of the model’s overall performance, category recognition capability, discrimination precision, and classification reliability. The specific formulas for these indicators are outlined in Equations (10)–(14):


$$\begin{array}{c} OA=\frac{\sum_{i=\text{1}}^{n}M_{ii}}{N} \end{array}$$ (10)

$$\begin{array}{c} PA_{i}=\frac{M_{ii}}{\sum_{i=\text{1}}^{n}M_{ij}} \end{array}$$ (11)

$$\begin{array}{c} UA_{i}=\frac{M_{ii}}{\sum_{j=\text{1}}^{n}M_{ji}} \end{array}$$ (12)

$$\begin{array}{c} Kappa=\frac{N\sum_{i=\text{1}}^{n}M_{ii}-\sum_{i=\text{1}}^{n}R_{i}C_{i}}{N^{\text{2}}-\sum_{i=\text{1}}^{n}R_{i}C_{i}} \end{array}$$ (13)

$$\begin{array}{c} F\text{1}=\text{2}\times \frac{PA\times UA}{PA+UA} \end{array}$$ (14)

In the formulas, N denotes the total number of samples; *Nij* indicates the number of samples whose true category is *i* but are predicted as category *j*; *Ni*. represents the total number of samples belonging to true category *i*; and *N.j* signifies the total number of samples predicted as category *j*. These formulas form the foundational calculation methods for constructing a confusion matrix and its associated accuracy metrics. In GEE, the corresponding confusion matrix can be directly obtained using errorMatrix(), from which additional evaluation indicators can subsequently be derived.

The overall data processing workflow of this study is illustrated in Fig 2.

**Fig 2:**
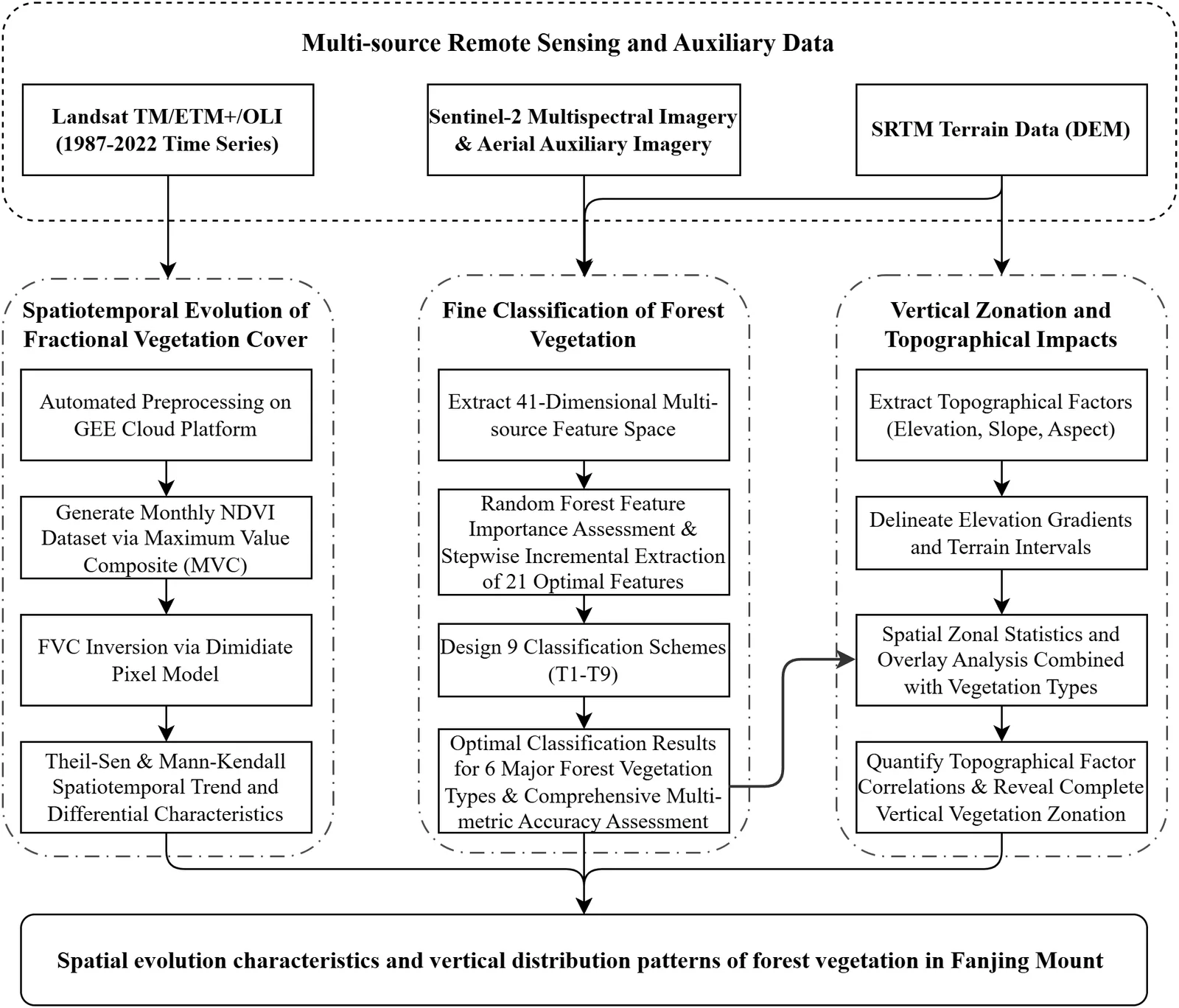
Overall technical workflow of this study.

## 3. Results and analysis

### 3.1. Analysis of vegetation cover changes in Fanjingountainm

To analyze vegetation coverage changes over the past 36 years, this study applied the SL190–2008 standard (Standards for Classification and Gradation of Soil Erosion), officially issued by the Ministry of Water Resources of China. This framework provides scientifically validated thresholds for assessing soil conservation capacity. Based on this standard and on-site characteristics, vegetation coverage was classified into five levels to characterize spatial heterogeneity: low (<30%), medium-low (30%–45%), medium (45%–60%), medium-high (60%–75%), and high (>75%).

#### 3.1.1. Spatial distribution characteristics of FVC in Fanjingountainm.

From 1987 to 2022, the vegetation coverage of Fanjing Mountain exhibited nonlinear changes and spatial heterogeneity. The initial fractional vegetation cover (FVC) in the base year of 1987 was recorded at 61.99%. Following periodic fluctuations, it peaked at 73.76% in 2016, reflecting an increase of 18.99%. However, this figure subsequently declined to 66.08% by 2022, resulting in an average annual growth rate of merely 0.18%. As illustrated in Fig 3, areas of high coverage are predominantly found in the central and southern hills, whereas lower coverage is more prevalent in the southeastern valleys. Notably, medium and high coverage accounts for approximately 32.57%, which significantly influences the evolution of the regional ecological pattern.

**Fig 3:**
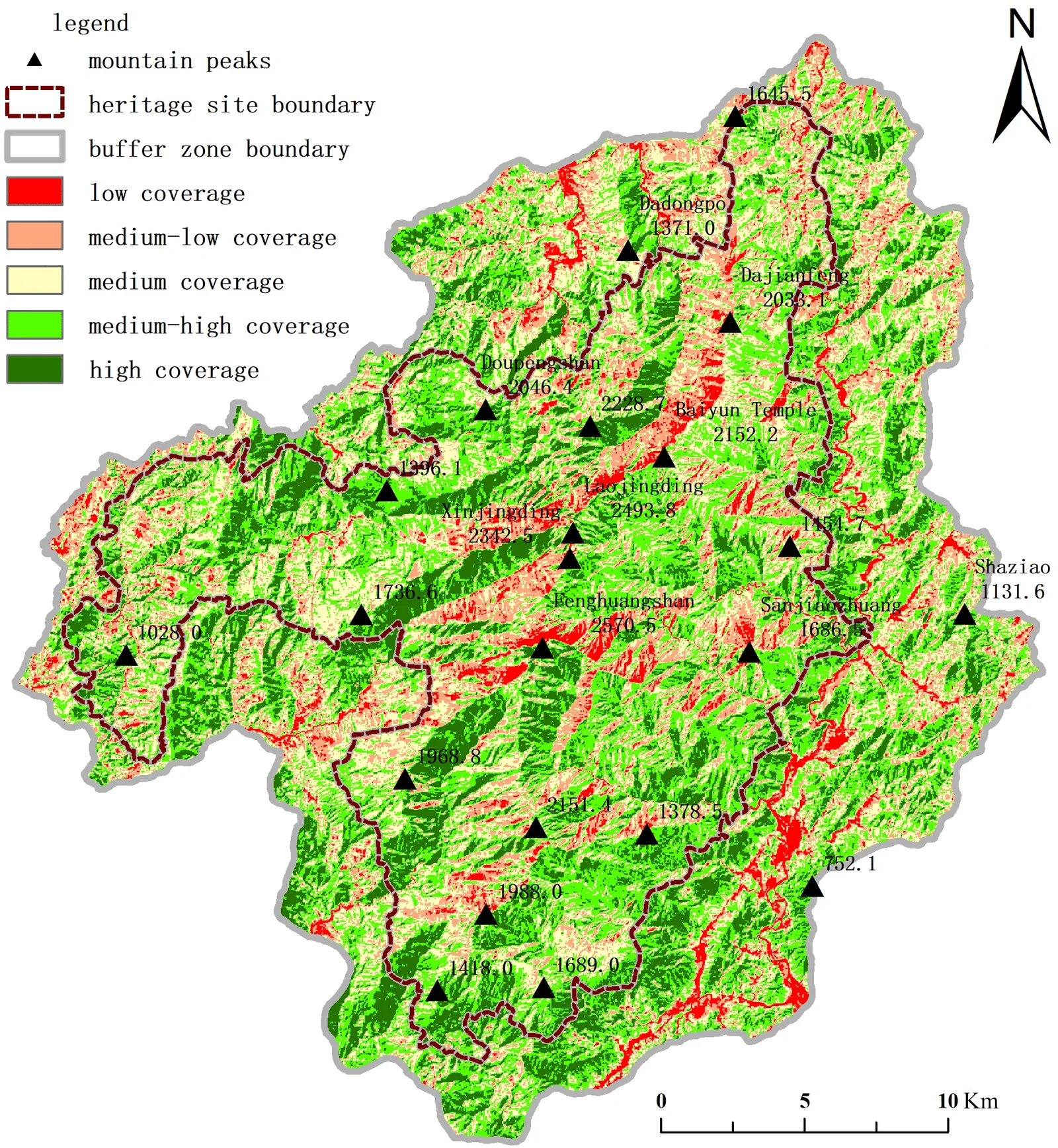
Spatial Distribution of the Mean FVC in Fanjing Mountain from 1987 to 2022. This figure was created with ArcGIS Pro (https://pro.arcgis.com/).

To comprehensively capture these ecological dynamics, continuous annual FVC datasets from 1987 to 2022 were generated; however, due to space constraints, only selected representative years are displayed. As illustrated in Fig 4, from 1987 to 2022, the vegetation coverage pattern of Fanjing Mountain has undergone a process characterized by degradation, recovery, and subsequent fluctuation adjustment. In 1987, the northwest, west, and southeast river valleys predominantly exhibited low coverage levels; conversely, the core area and eastern regions demonstrated a high coverage proportion of 38.89%, indicating a clear polarization. By 1995, the buffer zone had transformed into an area concentrated with low coverage while medium coverage became more evenly distributed across the landscape. This shift resulted in an overall homogenization of the vegetation structure. In 2001, significant vegetation degradation was observed as the area classified as low coverage expanded to 178.10 km^2^—accounting for 22.98%—with particularly severe degradation noted in the southwest region which emerged as an ecologically fragile zone. From 2008 onwards, signs of recovery became apparent; areas with medium-high and high coverage increased by approximately 19.56 km^2^ and 119.15 km^2^ respectively—an overall growth rate nearing 45%. The newly recovered areas were primarily located in the southeast. By 2016, ecological improvements persisted with medium-high and above coverage proportions surging to reach an impressive figure of 82.08%. A substantial continuous high-coverage area materialized within the core protection zone during this period. As we approached 2022, fluctuations in vegetation patterns were evident: specifically, there was a reduction of high-coverage areas in both central and northern sections by approximately 51.25%. Meanwhile, medium-high coverage ascended to constitute about 57.12%, establishing itself as the dominant category within this dynamic evolutionary stage of regional vegetation cover.

**Fig 4:**
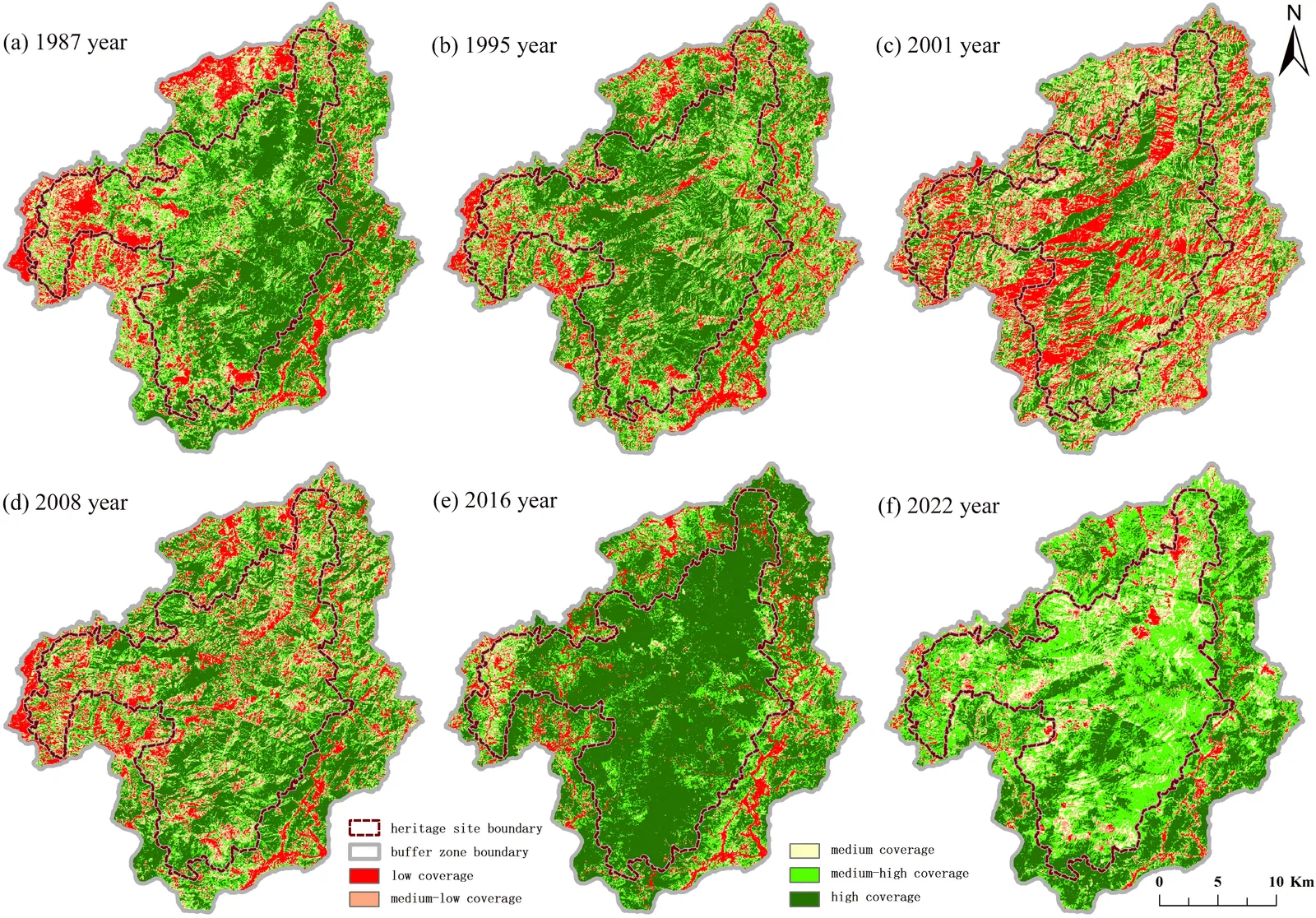
Spatial Distribution Characteristics of FVC at Different Levels in Fanjing Mountain from 1987 to 2022. This figure was created with ArcGIS Pro (https://pro.arcgis.com/).

#### 3.1.2. Interannual Variation Characteristics of FVC in Fanjing Mountain.

Fig 5 illustrates that the average vegetation coverage (FVC) of Fanjing Mountain from 1987 to 2022 has demonstrated distinct phased evolutionary characteristics. Utilizing the moving window method, the years 2007 and 2013 were identified as significant turning points in this trajectory. Spatially, these evolutionary turning points exhibit regional differences: the core protected areas and high-altitude zones maintained relatively stable high coverage due to long-term restricted human access, whereas the overall trend shifts were primarily driven by significant vegetation changes in the lower-altitude edge regions. The overall process can be categorized into three stages: fluctuating decline (1987–2007), oscillating adjustment (2008–2013), and ecological leap (2014–2022). Over the past 36 years, the FVC has generally exhibited an upward fluctuation, with an average growth rate of 4.151% per decade. The lowest recorded value occurred in 2007 at 49.64%, while the average during the study period was found to be 59%, with interannual fluctuations ranging from 50% to 74%.

**Fig 5:**
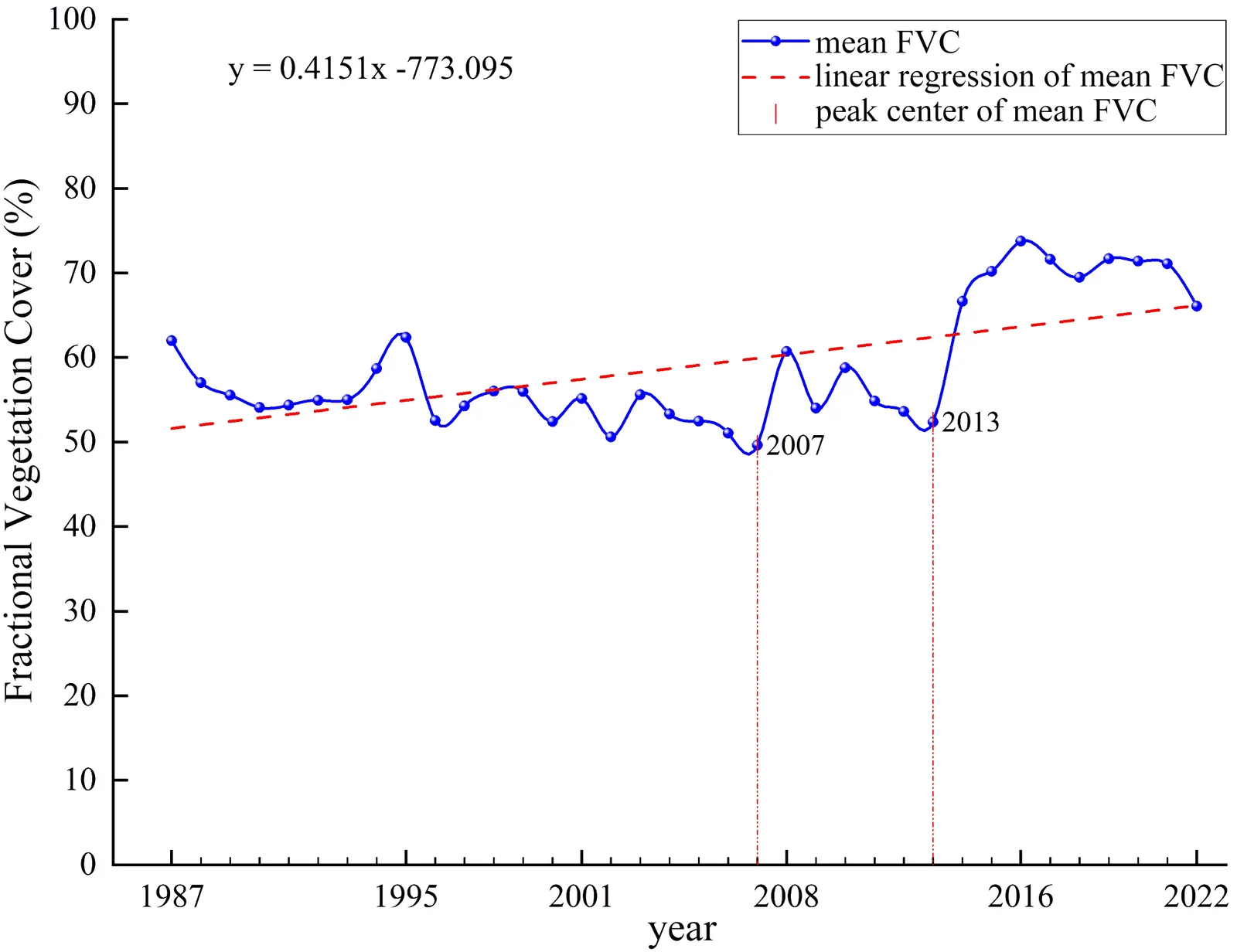
Mean Changes of FVC in Fanjing Mountain from 1987 to 2022.

From 1987 to 2007, the average Fractional Vegetation Cover (FVC) remained approximately at 55%, exhibiting a continuous downward trend with a cumulative decline of 12.35%. Following 2007, the average FVC increased to 64% as early macroscopic ecological engineering (such as the Grain for Green program) began to mitigate vegetation degradation. However, the region. However, the region experienced significant fluctuations from 2008 to 2013, reaching a low of 52.38%, reflecting the vulnerability of the early recovering ecosystem to natural climate disturbances, notably the severe ice and snow disaster in 2008. Since 2014, it has entered an ecological restoration phase, with FVC showing a notable increase to 66.65%, coinciding with and directly driven by strict conservation policies initiated during the World Heritage application in 2013. It peaked historically at 73.76% in 2016, characterized by dominant high coverage levels. Although there was a slight decrease to 66.08% in 2022, this figure remains significantly higher than early levels, indicating that the year 2007 marked a critical turning point resulting from the interplay of natural and human factors, and that an evident trend of ecological recovery has been observable since 2013.

#### 3.1.3. The characteristics of FVC spatial changes in Fanjinguntainmo.

This study integrates Theil-Sen trend estimation with the Mann-Kendall significance test to quantitatively analyze the spatio-temporal variation characteristics of vegetation coverage (FVC) in Fanjing Mountain National Nature Reserve from 1987 to 2022. This methodology effectively assesses the intensity, direction, and significance level of trends. A Z value exceeding 1.65, 1.96, and 2.58 indicates statistical significance at the 90%, 95%, and 99% confidence levels, respectively. A positive Z value signifies improvement, whereas a negative Z value denotes degradation (specific classifications are detailed in Table 4).

**Table 4:** Significance Levels of Trends in Vegetation Coverage Fraction (FVC).

β	Z	Trend Categories	Trend characteristics
β>0	2.58 < Z	4	Significantly improved
	1.96 < Z ≤ 2.58	3	Significantly improved
	1.65 < Z ≤ 1.96	2	Significantly improved
	Z ≤ 1.65	1	No significant improvement
β=0	Z	0	basically stable
β<0	Z ≤ 1.65	−1	insignificant degradation
	1.65 < Z ≤ 1.96	−2	Slight degradation
	1.96 < Z ≤ 2.58	−3	Significant degradation
	2.58 < Z	−4	Extremely significant degradation

Fig 6 illustrates that over the span of 36 years, the FVC exhibited a general improvement, with an enhanced area of 322.82 km^2^ (41.65%). This includes a highly significant improvement area of 80.71 km^2^ (10.41%), predominantly concentrated in the southern, western, and southeastern regions; alongside a significant improvement area of 55.44 km^2^ (7.15%), which is distributed in a more scattered manner. The total degradation area amounted to 134.28 km^2^ (17.32%), with a highly significant degradation zone covering 10.49 km^2^(1.35%), primarily located in the eastern region at elevations between 800–1000 m.The central core area remained relatively stable, with the most pronounced improvements observed in the south. Analyzing inter-annual evolution characteristics reveals that 2007 served as a pivotal turning point for vegetation change, leading to the division of the study period into two distinct stages: from 1987 to 2007 and from 2007 to 2022 (Fig 7). During the period from 1987 to 2007, degradation was predominant, encompassing an area of 345.18 km^2^(44.53%) and mainly concentrated in eastern and southeastern areas; notably, highly significant degradation reached up to 39.16 km^2^ (5.05%). In contrast, improved areas totaled approximately167.06 km^2^ (21.55%), forming a belt-shaped distribution across southwest-northwest orientations; however, only about5.14km^2^ (0.66%) demonstrated highly significant improvement.

**Fig 6:**
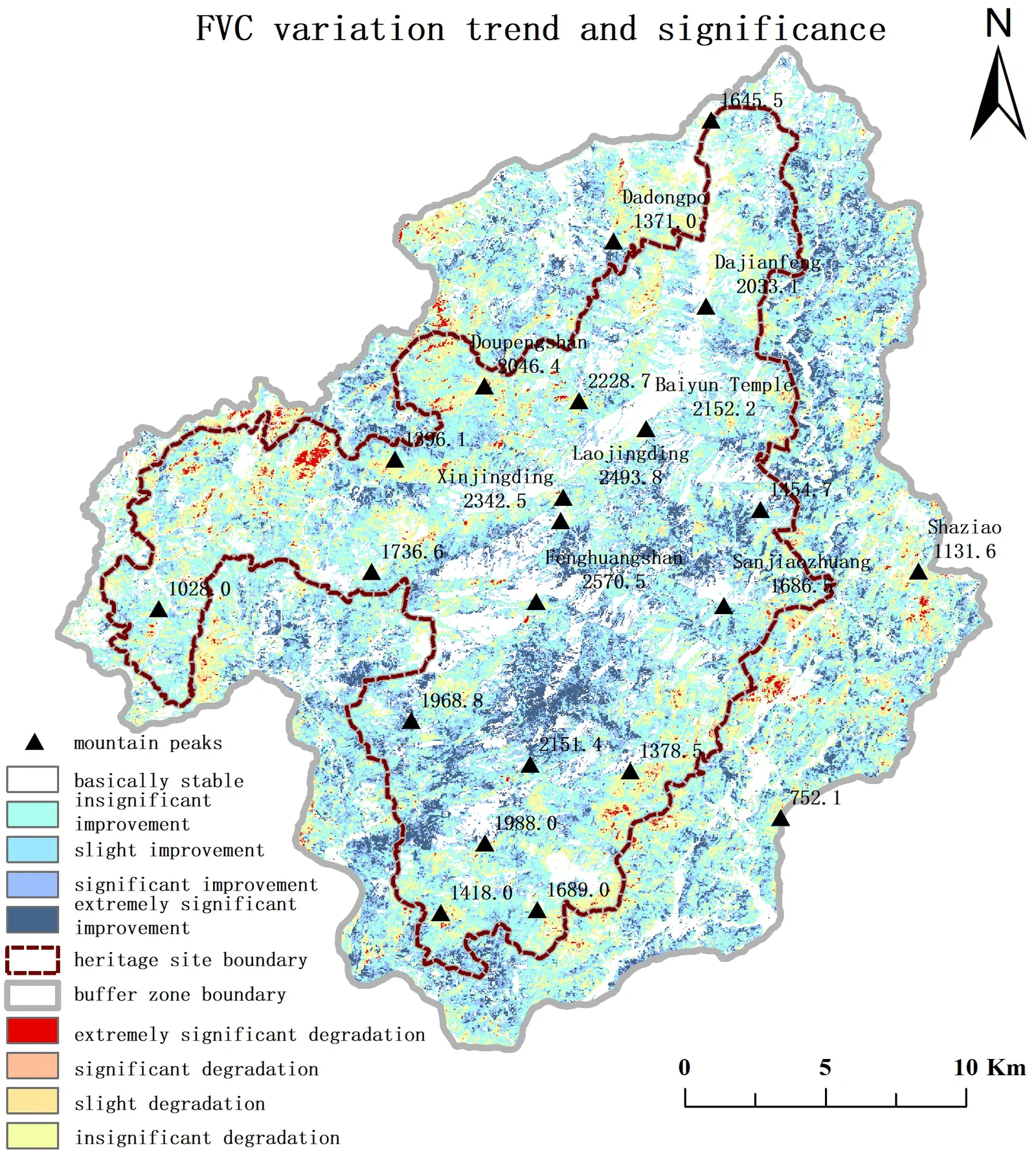
Spatial Variation Trend and Significance of Vegetation Coverage in Fanjing Mountain from 1987 to 2022. This figure was created with ArcGIS Pro (https://pro.arcgis.com/).

**Fig 7:**
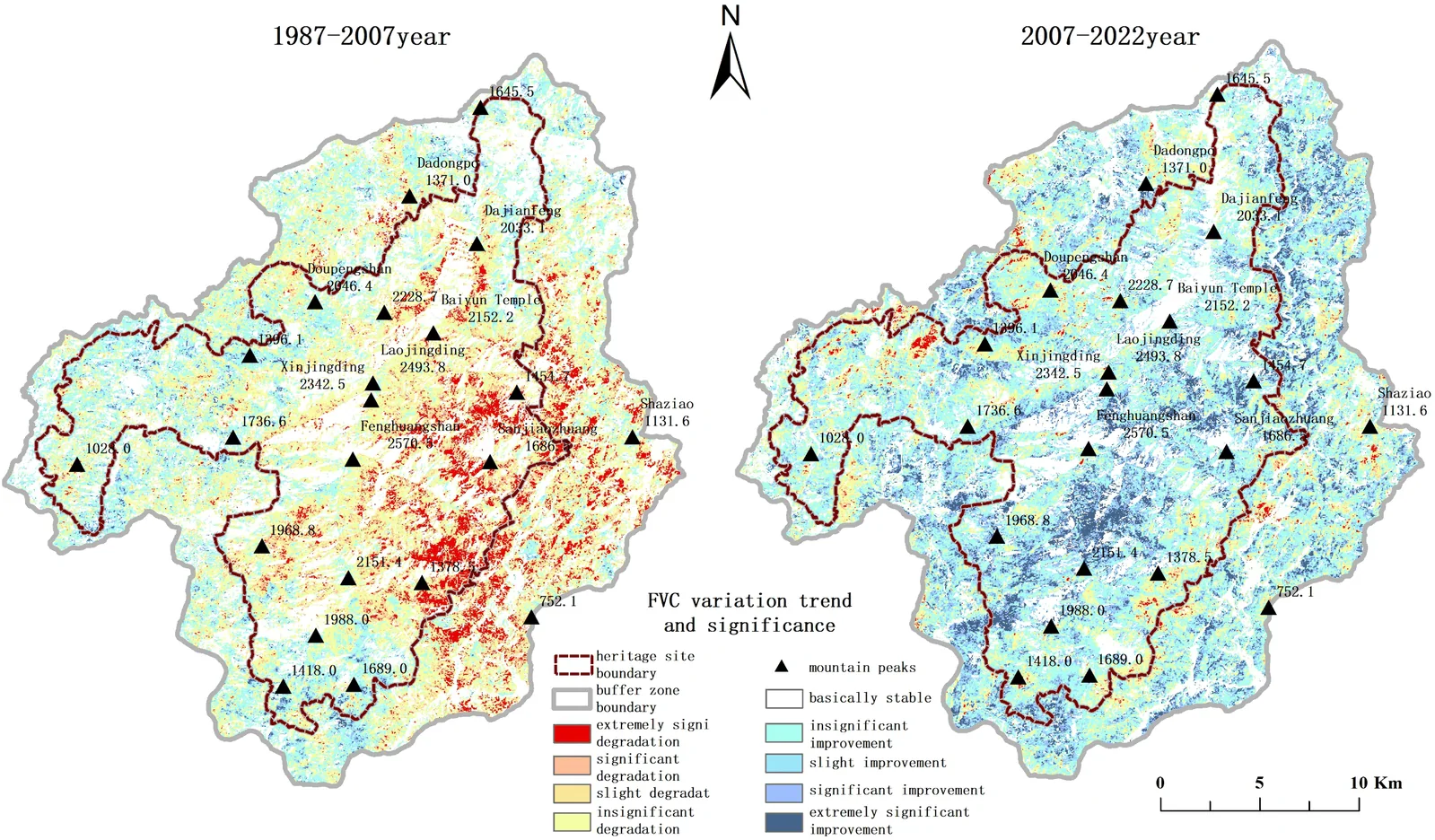
Spatial Variation Trends and Significance of Vegetation Coverage in Different Time Periods in Fanjing Mountain. This figure was created with ArcGIS Pro (https://pro.arcgis.com/).

From 2007 to 2022, a notable trend of improvement was observed, with the area exhibiting enhancement increasing to 442.98 km^2^ (57.15%). This improvement transitioned from being concentrated in specific locations to becoming more widespread across the entire region. The area demonstrating extremely significant improvement reached 57.45 km^2^, primarily distributed in the central and southern sections of the heritage site as well as in the northeastern part of the buffer zone. Conversely, areas showing insignificant improvement constituted the largest proportion at 31.29%, indicating a gradient pattern of enhancement extending from the heritage site into the buffer zone.The extent of degradation decreased to 135.79 km^2^, reflecting a reduction of 60.63% compared to previous periods. The remaining degraded areas have become increasingly marginalized and fragmented over time. Overall regional stability has improved, with basic stability encompassing an area of 196.36 km^2^ (25.33%), predominantly situated within the high-altitude core region.

### 3.2. Forest vegetation classification

#### 3.2.1. Vegetation classification system and sample construction.

Based on the ecological characteristics of the Fanjing Mountain forest, this study established an integrated data system encompassing “ground-air-space.” The surface coverage was categorized into six distinct types: shrub meadow, bamboo forest, evergreen broad-leaved forest, coniferous forest, deciduous broad-leaved forest, and others. This classification system was developed in conjunction with the “Vegetation Map of the People’s Republic of China (1:1,000,000)” and supported by field investigations.Sample data collection utilized second-class surveys of forest resources alongside aerial photography and high-resolution images from Google Earth. A strategy that combined GIS spatially balanced sampling with stratified sampling was employed to generate a total of 3,821 valid samples. These samples were subsequently divided into training and validation sets at an 80:20 ratio to ensure both spatial independence and temporal consistency.

In the realm of remote sensing feature extraction, the system integrates a total of 41 indicators, encompassing Sentinel-2 multispectral bands, vegetation indices, red-edge indices, texture features, and terrain factors to construct a comprehensive joint feature set that includes spectral, spatial, and terrain dimensions. Among these indicators, 10 key bands from Sentinel-2 are selected to develop a spectral response model. For vegetation indices, 12 representative metrics such as NDVI (Normalized Difference Vegetation Index), EVI (Enhanced Vegetation Index), RVI (Ratio Vegetation Index), ARVI (Atmospherically Resistant Vegetation Index), SAVI (Soil Adjusted Vegetation Index), and KNDVI (Kernel Normalized Difference Vegetation Index) are computed to enhance the differentiation among various vegetation classes. A detailed categorization of all 41 feature variables utilized in this study is provided in Table 5.

**Table 5:** Full list of variables used in the classification model.

Feature Category	Feature Name	Description
Multispectral Bands (10)	B2 (Blue)	Sensitive to water bodies and atmospheric scattering.
	B3 (Green)	Highlights peak vegetation reflection in the visible spectrum.
	B4 (Red)	Primary band for chlorophyll absorption.
	B5 (Red Edge 1)	Sensitive to early red-edge transition and plant stress.
	B6 (Red Edge 2)	Core red-edge band for chlorophyll content estimation.
	B7 (Red Edge 3)	Reflects late red-edge canopy structural variations.
	B8 (NIR)	Strongly reflects vegetation biomass and cell structure.
	B8A (Narrow NIR)	Narrow NIR band avoiding water vapor absorption.
	B11 (SWIR 1)	Sensitive to vegetation and soil moisture content.
	B12 (SWIR 2)	Captures dead vegetation and geological features.
Vegetation Indices (12)	NDVI	Indicates general vegetation greenness and biomass.
	EVI	Minimizes soil and atmospheric influences in high biomass areas.
	DVI	Indicates vegetation amount with sensitivity to soil background.
	RVI	Enhances contrast between vegetation and background.
	ARVI	Resists atmospheric aerosol effects on vegetation signals.
	GLI	Measures green leaf density based on visible bands.
	RG	Revealing ratio changes between leaf chlorophyll and anthocyanin.
	MNDWI	Enhances water bodies while suppressing built-up land noise.
	BSI	Quantifies bare soil exposure and soil variations.
	NDBI	Highlights urban and built-up land features.
	SAVI	Corrects for soil background in low vegetation cover areas.
	KNDVI	Reflects photosynthetic activity biomass and growth trends.
Red-edge Indices (7)	NDVIre1	Red-edge based NDVI utilizing B5 for plant stress.
	NDVIre2	Red-edge based NDVI utilizing B6 for chlorophyll content.
	NDVIre1n	Narrow red-edge NDVI utilizing B8A and B5.
	NDVIre2n	Narrow red-edge NDVI utilizing B8A and B6.
	CIre	Estimates total canopy chlorophyll content.
	MTCI	Sensitive to high chlorophyll content and leaf area index.
	MCARI	Measures depth of chlorophyll absorption resisting background.
Texture Features (8)	Angular Second Moment (ASM)	Measures image uniformity and structural order.
	Contrast	Measures local variations and image sharpness.
	Correlation	Measures linear dependency of local gray levels.
	Variance	Measures heterogeneity and gray level distribution.
	Inverse Difference Moment (IDM)	Measures local homogeneity and image smoothness.
	Sum Average	Reflects overall image brightness distribution.
	Sum Variance	Measures the dispersion of sum of gray levels.
	Entropy	Indicates image complexity and information content.
Topographic Features (4)	Elevation	Absolute altitude affecting spatial distribution of vegetation.
	Slope	Steepness or degree of terrain inclination.
	Aspect	Compass direction the terrain faces.
	Hillshade	Simulated terrain illumination and shadows.

Additionally, texture features are derived using the gray-level co-occurrence matrix in conjunction with terrain variables including slope, aspect, and elevation; this approach provides multi-dimensional input support for subsequent high-precision classification models.Through random forest feature importance analysis (Fig 8), it is observed that the contribution of terrain factors—namely DEM (Digital Elevation Model), slope, and aspect—is significantly greater than that of traditional vegetation indices. The stepwise incremental method is employed to identify the optimal combination of features: when the number of features increases to 21—which encompasses terrain data along with vegetation indices, red-edge spectra, texture attributes, and spectral bands—the overall accuracy (OA) peaks at 77.88%, while the Kappa coefficient reaches 0.71 (Fig 9).

**Fig 8:**
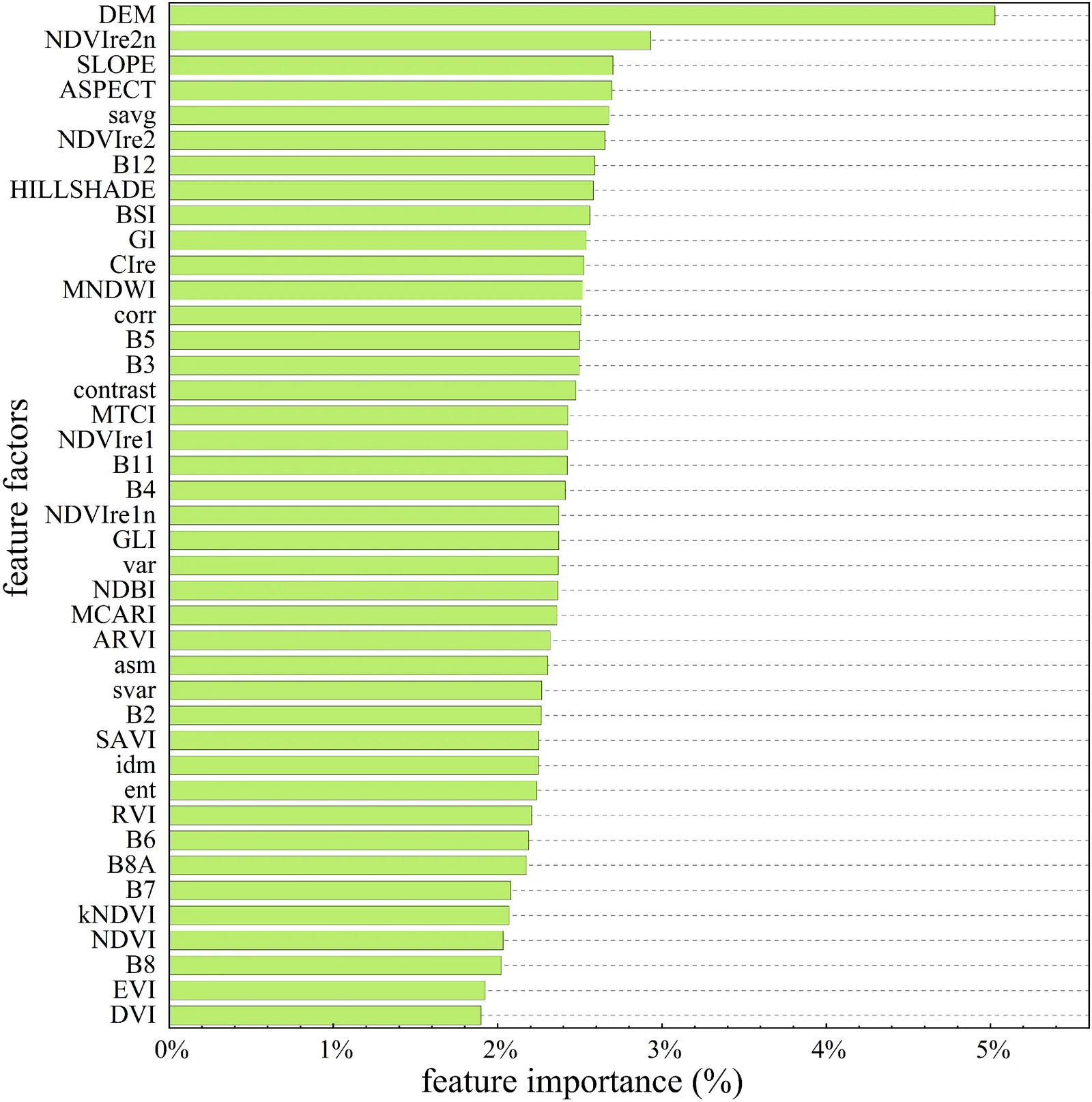
Random Forest Feature Importance Ranking.

**Fig 9:**
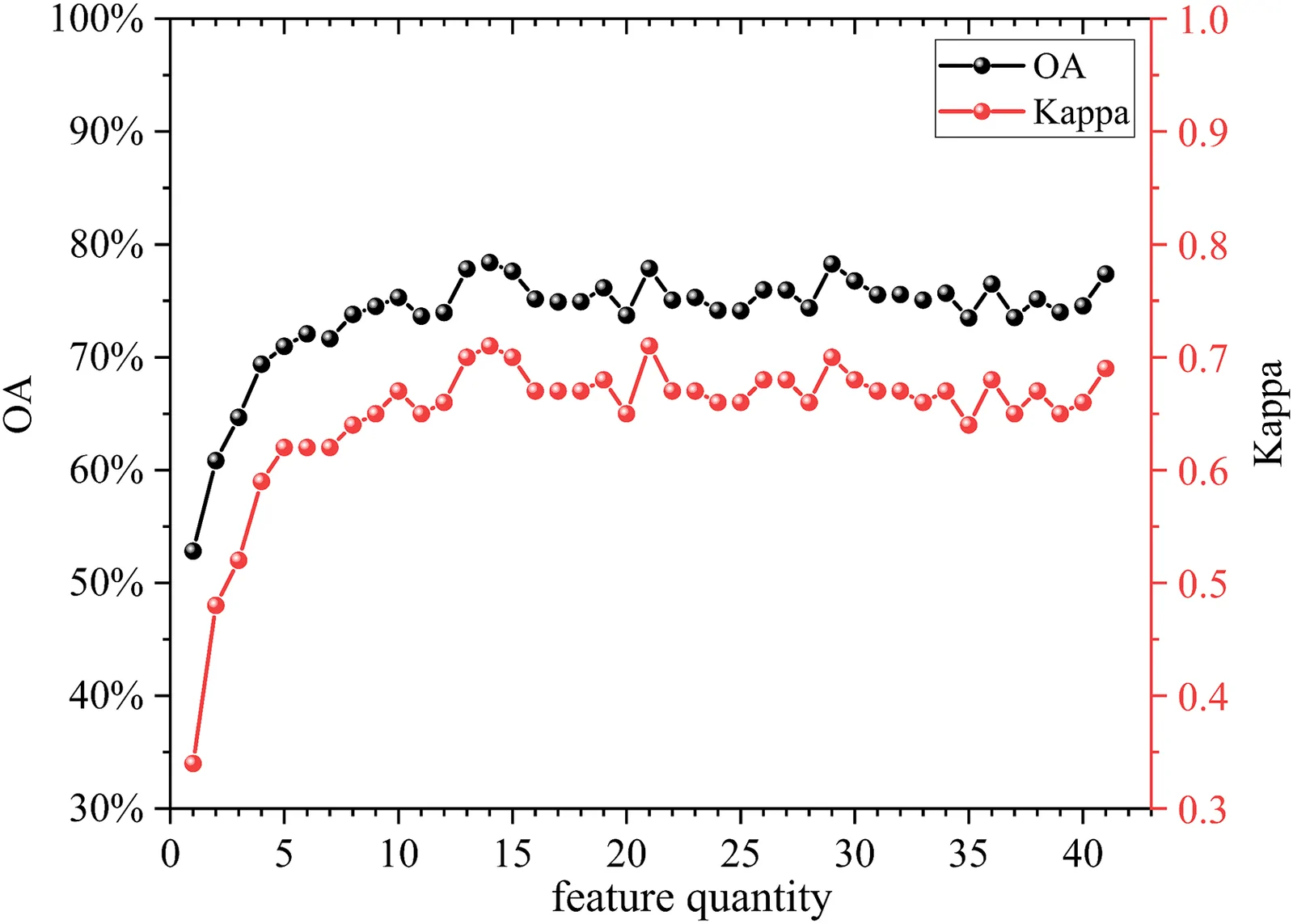
Relationship between Feature Quantity and Classification Accuracy.

#### 3.2.2. Classification experiment.

Nine feature combination schemes (T1 - T9) were developed in this research, progressively incorporating multispectral, topographic, vegetation index, red-edge index, and texture features to assess the contribution of each feature to classification accuracy. Unlike unsupervised dimensionality reduction techniques such as Factor Analysis (FA), which generate artificial components through linear combinations and obscure the original physical and ecological meanings of remote sensing variables, this progressive evaluation preserves the specific interpretability essential for mountain vegetation analysis. Furthermore, rather than relying solely on a simple classical classification method, this study designed the 9-experiment framework as a systematic ablation study. By establishing a simple multispectral classification as the baseline (T1), this methodology justifies the advantages of the optimized multi-source model (T9) by quantitatively evaluating the independent, synergistic, and redundant effects of incrementally added features. The classification model employed the random forest algorithm on the Google Earth Engine platform, with a training set and validation set divided in an 80:20 ratio. Among these schemes, T1 served as the baseline configuration utilizing only multispectral bands; T2 introduced topographic features that significantly enhanced the identification capability of shrub meadows; T3 added vegetation indices but demonstrated limited effectiveness in complex mountainous regions; T4 incorporated the red-edge index, which improved the identification of evergreen broad-leaved forests; while T5 integrated all features—despite a substantial volume of information—the overall classification performance did not show significant improvement. Schemes T6 through T8 tested the independent effects of vegetation indices, red-edge indices, and texture features respectively. The results indicated that relying on a single feature was insufficient to meet classification requirements. Ultimately, scheme T9 achieved optimal classification performance by eliminating redundant features. The experimental schemes along with their respective feature combinations are presented in Table 6.

**Table 6:** Classification scheme.

Experimental plan	Feature combination	Note
T1	Multispectral bands	Using only multispectral bands, baseline scheme
T2	Multispectral bands + terrain features	Enhancing the identification of vegetation distribution in complex terrain areas
T3	Multispectral bands + terrain features + vegetation indices	Improving vegetation health and density differentiation
T4	Multispectral bands + terrain features + vegetation indices + red-edge spectral indices	Strengthen the discrimination of high-density vegetation areas
T5	Multispectral bands + terrain features + vegetation indices + red-edge spectral indices + texture features	Integrate spatial structure information and test the scheme with the maximum information volume.
T6	Multispectral bands + vegetation indices	Without considering the terrain, only analyze the contribution of the vegetation index.
T7	Multispectral bands + red-edge spectral index	Only increase the red-edge spectral index and test its independent effect.
T8	Multispectral bands + texture features	Test only with texture features and spatial structure features added.
T9	Optimal feature combination	Eliminate redundant features

#### 3.2.3. Analysis of classification results.

As illustrated in Fig 10, the classification results for each scheme reveal that the T1 scheme exhibits a pronounced “ salt-and-pepper noise” phenomenon alongside relatively low classification accuracy. The T2 scheme, which incorporates topographic features, significantly enhances classification performance, particularly in identifying shrub meadows. Conversely, the T3 scheme demonstrates a decline in classification effectiveness after adding vegetation indices; this may be attributed to redundancy in spectral information. The T4 scheme improves recognition accuracy for evergreen broad-leaved forests by integrating red-edge index. However, the T5 scheme—despite its comprehensive integration of all features—does not yield a significant enhancement in classification outcomes and incurs a comparatively high computational cost. The schemes from T6 to T8 evaluate the independent effects of vegetation indices, red-edge index, and texture features respectively; however, their classification results are unsatisfactory. A comprehensive analysis indicates that the T9 scheme achieves optimal classification performance with consistent results and a marked reduction in the “ salt-and-pepper noise” phenomenon. Furthermore, it clearly delineates the distribution characteristics of various vegetation types.

**Fig 10:**
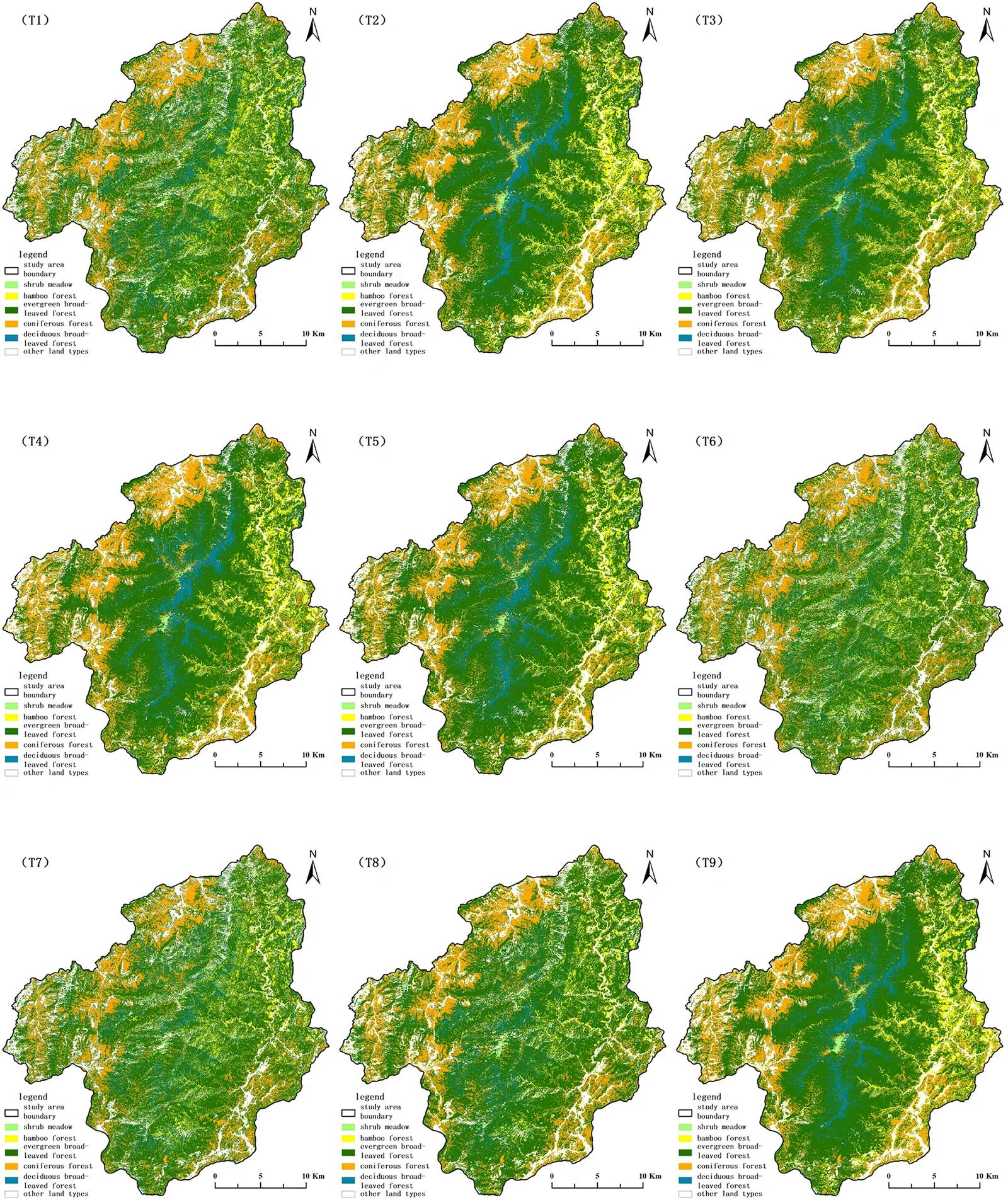
Classification Results of Each Scheme. This figure was created with ArcGIS Pro (https://pro.arcgis.com/).

The assessment of classification accuracy, as illustrated in Figs 11 and 12, indicates that the overall accuracy (OA) of the T9 scheme is 77.88%, with a Kappa coefficient of 0.71, reflecting a significant level of consistency. Among various vegetation types, the producer’s accuracy (PA) for shrub meadows reaches an impressive 1.00, while the user’s accuracy (UA) stands at 0.83 and the F1 score is recorded at 0.90, demonstrating optimal performance; conversely, the PA for coniferous forests is only 0.44, with a UA of 0.74 and an F1 score of 0.55, indicating relatively poor recognition results.Furthermore, the classification accuracy for other vegetation types within the T9 scheme has also shown improvement, underscoring the effectiveness of collaboratively optimizing multi-source features. To provide a detailed quantitative evaluation of this optimal performance, the confusion matrix for the T9 scheme is presented in Fig 13. In conclusion, the T9 scheme significantly enhances forest vegetation classification accuracy by judiciously integrating multi-source remote sensing features, thereby providing a reliable data foundation for vertical belt spectrum analysis in the Fanjing Mountain area.

**Fig 11:**
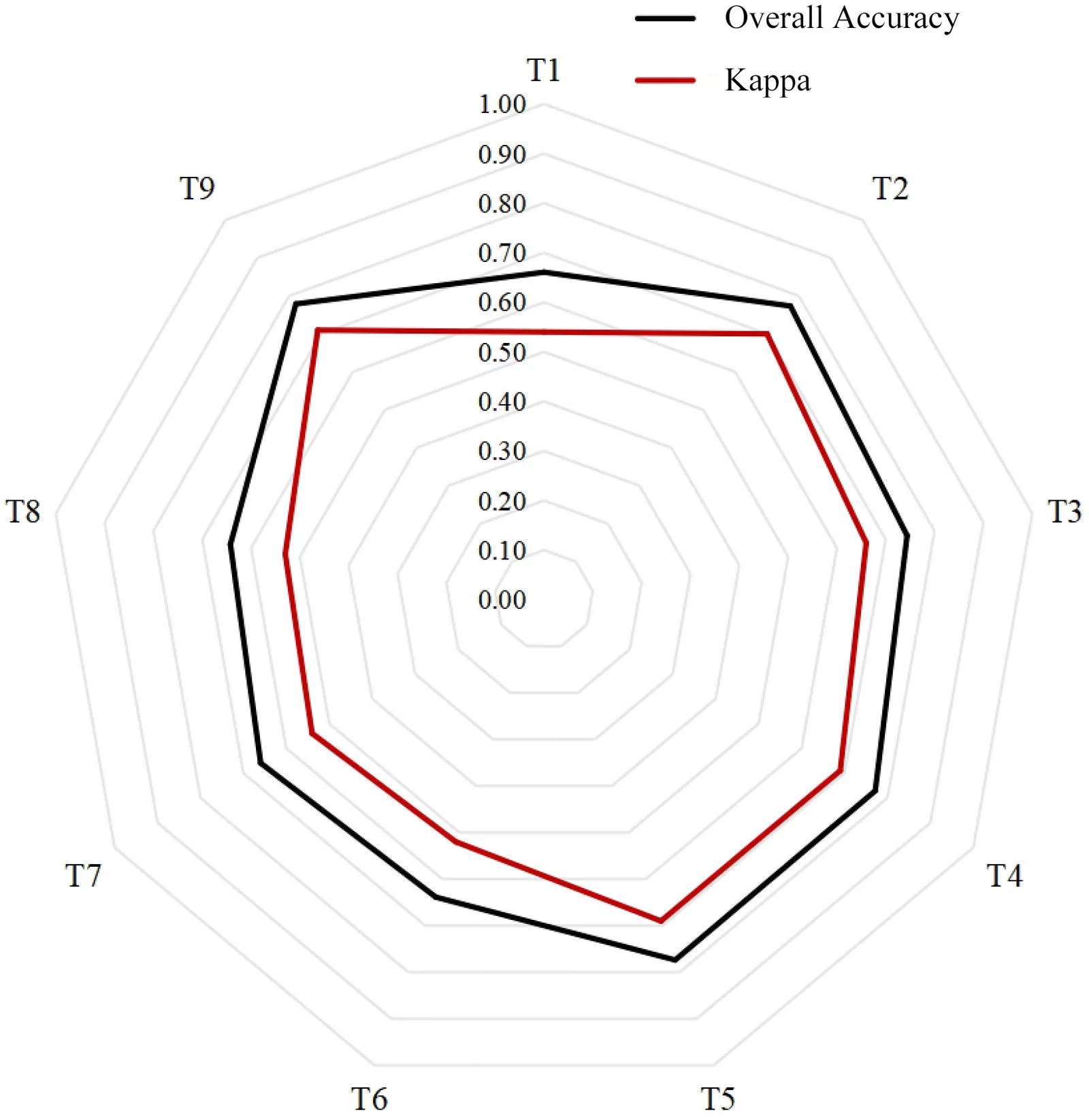
OA and Kappa Coefficients of Each Scheme.

**Fig 12:**
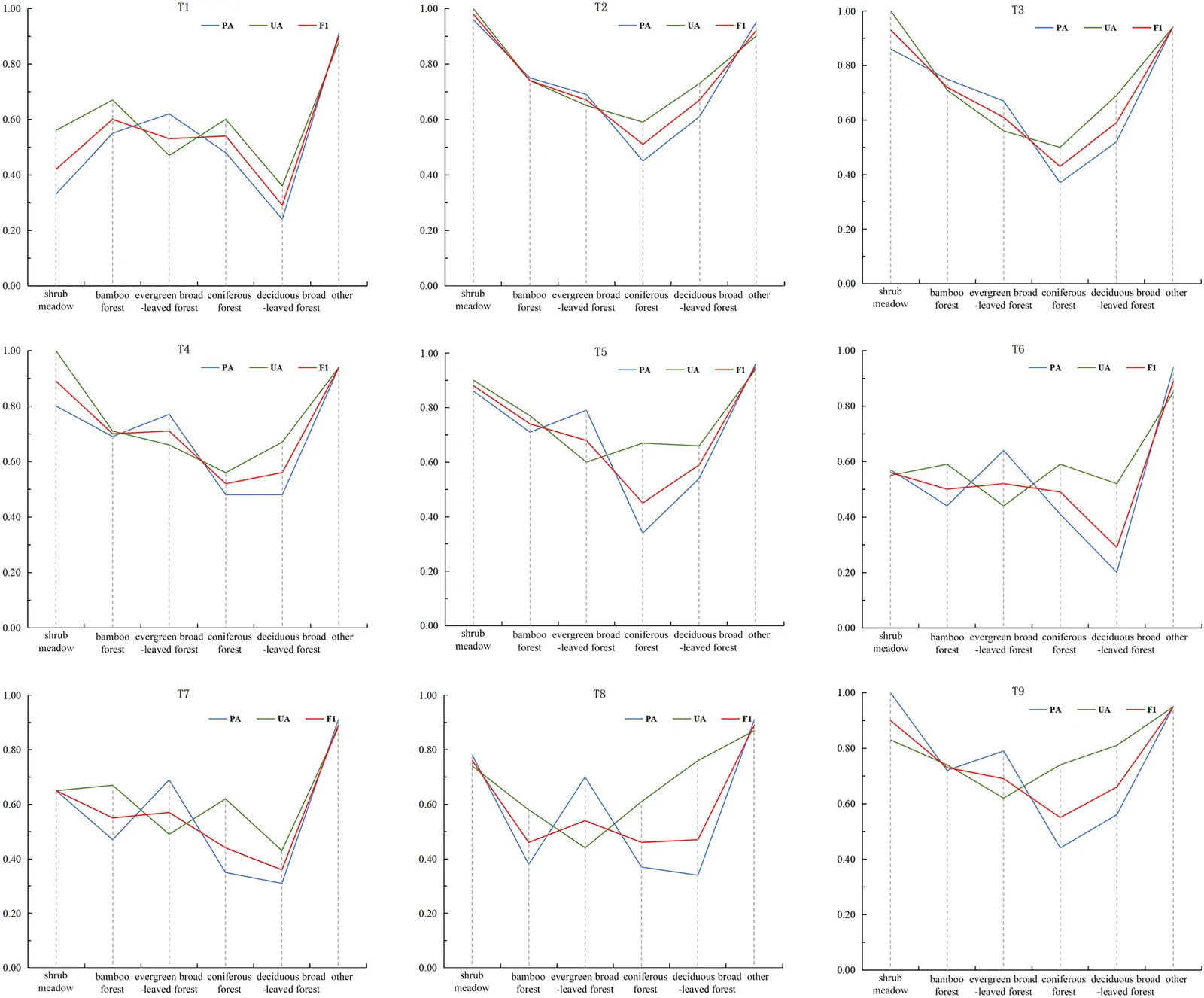
Classification Accuracy of Each Scheme.

**Fig 13:**
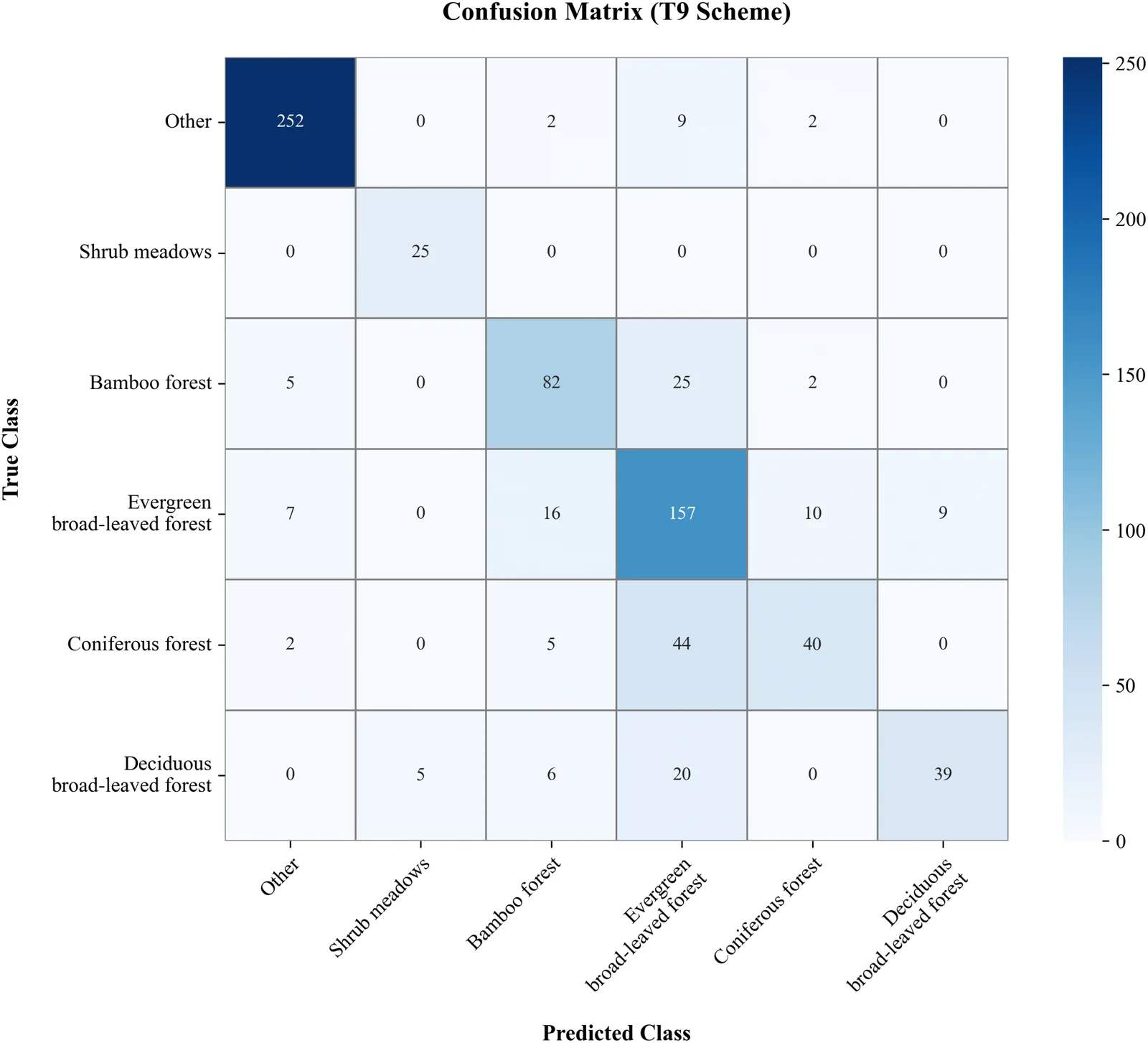
Confusion matrix of the optimal classification scheme (T9).

### 3.3. Analysis of altitudinal vegetation zones

Based on the T9 classification results, non-forest types were excluded, and the vertical distribution characteristics of various vegetation types were analyzed in conjunction with DEM data. The study area was categorized into six altitude intervals: < 700m, 700m - 1300m, 1300m - 1900m, 1900m - 2100m, 2100m - 2350m, and 2350m - 2565m. As illustrated in Fig 14, the findings indicate that at lower altitudes (<1300m), evergreen broad-leaved forests are predominant, comprising approximately 50% within the range of 700m to 1300m; bamboo forests are primarily concentrated below an elevation of 1100m. At medium altitudes (1300m - 2100m), evergreen broad-leaved forests account for about 80% within the interval of 1300m to1900 m. In contrast, deciduous broad-leaved forests increase to represent approximately38% in the range of 1900m to 2100m while coniferous forests reemerge. At higher altitudes (>2100m), shrub meadows constitute around 65% within the interval from 2350m to 2565m. Notably, coniferous forests exhibit a “double-peak distribution,” being concentrated at both low and high elevations.The average altitude associated with each vegetation type reflects its ecological adaptability: shrub meadows thrive above 2400m; coniferous forests display a wide distribution between 700m and 2400m; deciduous broad-leaved forests predominantly occupy medium to high altitudes; evergreen broad-leaved forests are mainly found between 750m and 1550m and bamboo forests are located below 1100m influenced by water sources and human disturbances. A gradient succession sequence is established from low to high altitudes as follows: bamboo forests (<1100m), evergreen broad-leaved forests (750–1550m); deciduous broad-leafed forest(1800m–2100m); coniferous forest(700–2400m) and shrub meadow (>2400m).

**Fig 14:**
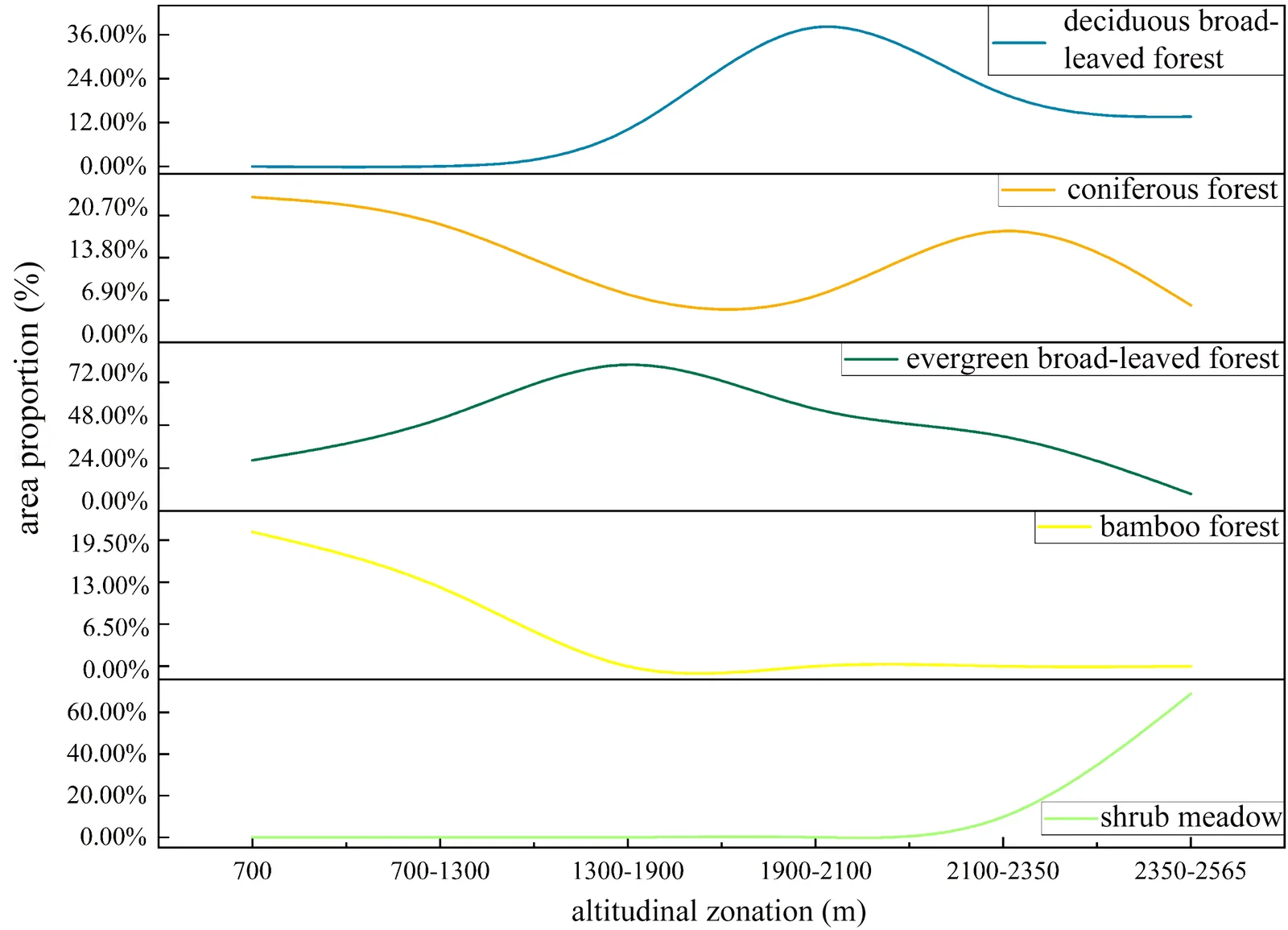
Trend of Area Proportion of Different Vegetation Types with Altitude Variation.

## 4. Discussion

In this study, the spatiotemporal evolution patterns of vegetation cover and the vertical distribution characteristics of forest vegetation in Fanjing Mountain were systematically investigated through the integration of multi-source remote sensing data and refined classification techniques. Nevertheless, certain limitations persist, necessitating further exploration and refinement.

### 4.1. Limitations of data sources

The study employed Landsat series and Sentinel-2 multispectral images. However, the spatial resolution of these datasets may be insufficient to fully capture fine-scale vegetation distribution details in complex mountainous terrains. Although sub-meter-resolution aerial imagery was accessible, its limited spatial coverage precluded a comprehensive depiction of vegetation distribution across the entire reserve, thereby affecting classification accuracy in certain localized areas. Future research should consider integrating higher-resolution remote sensing data with more extensive ground surveys to enhance classification precision further.

### 4.2. Limitations of classification methods

Although effective, the random forest model is fundamentally a statistical algorithm reliant on feature selection and sample quality. Despite optimizing feature combinations, issues such as feature redundancy or information loss may still affect model generalization. Distinguishing fine-grained vegetation subtypes in complex mountainous terrains remains highly challenging. The 77.88% overall accuracy aligns with applications in similar environments, which typically report accuracies ranging from 75% to 85% even when employing advanced algorithms or high-resolution UAV imagery [25–27]. This performance was primarily constrained by high inter-class spectral similarity, severe topographic shadow effects [28], and the poor recognition of coniferous forests (producer accuracy of 0.44), which is attributed to their low visible-band reflectance and complex structural characteristics. Furthermore, categorizing vegetation into six broad classes may overlook internal heterogeneity, as broadleaf forests contain undelineated subclasses that limit classification precision. Future work could explore deep learning models, such as convolutional neural networks (CNNs), to process high-dimensional remote sensing data and capture more complex spectral and spatial features.

## 5. Conclusion

This study presents a quantitative analysis of the spatio-temporal changes in Fractional Vegetation Cover (FVC) on Fanjing Mountain from 1987 to 2022, elucidating the evolutionary stages and spatial heterogeneity patterns of regional vegetation coverage. By integrating multi-source remote sensing data, we conducted a forest vegetation classification experiment and established a high-precision classification system. Building upon this foundation, we performed vertical belt spectrum vertical belt spectrum analysis to systematically uncover the elevation gradient characteristics of forest vegetation on Fanjing Mountain. The main conclusions are as follows:

(1) Over the past 36 years, Fanjing Mountain has exhibited a notable improvement in vegetation coverage, characterized by significant ecological recovery. From 1987 to 2022, the average annual growth rate of Forest Vegetation Coverage (FVC) on Fanjing Mountain was recorded at 0.18%, revealing a three-stage pattern of fluctuating decline, oscillating adjustment, and ecological leap. The year 2007 marked a critical turning point from degradation to recovery, driven by the interplay of early ecological engineering and natural climate fluctuations, whereas the subsequent ecological leap since 2013 was directly attributed to strict conservation policies during the World Natural Heritage application. The Theil-Sen trend analysis indicated that 41.65% of the area demonstrated an improving trend in FVC, particularly in the southern region and core areas, underscoring the effectiveness of implemented ecological protection policies. Spatially, while core protected areas maintained relatively stable high coverage, the overall turning characteristics of the region were primarily dominated by significant vegetation changes in lower-altitude edge areas.
(2) The integration of remote sensing features—including terrain data, red-edge indices, and texture characteristics (T9 scheme)—significantly enhanced forest classification accuracy. This optimized combination of multi-source remote sensing features markedly improved the identification capabilities for various forest types. The overall accuracy achieved with the T9 scheme reached 77.88%, accompanied by a Kappa coefficient of 0.71; notably, shrub meadows and evergreen broad-leaved forests were identified with exceptional precision. Furthermore, it was observed that terrain factors contributed more significantly than traditional vegetation indices, highlighting the importance of synergistic effects between terrain and structural information within complex mountainous environments.
(3) Building upon high-precision classification results, an initial vertical belt spectrum pattern for forest vegetation was established. By integrating T9 classification outcomes with Digital Elevation Model (DEM) data, distinct altitudinal distributions of forest types were identified—reflecting the predominant influence of thermal zones on determining vegetation types. This research provides both a scientific foundation and spatial basis for future studies on vertical belt spectrum patterns and ecological zone delineations.
